# Comparison of Different Types of Corneal Foreign Bodies Using Anterior Segment Optical Coherence Tomography: A Prospective Observational Study

**DOI:** 10.1155/2020/9108317

**Published:** 2020-08-11

**Authors:** Tao Wang, Lei Zhong, Shiyi Yin, Tiancheng Bao, Jiezheng Yang, Ting Wang, Shiqi Ling

**Affiliations:** ^1^Department of Ophthalmology, Third Affiliated Hospital of Sun Yat-Sen University, Guangzhou, Guangdong, China; ^2^State Key Laboratory of Ophthalmology, Zhongshan Ophthalmic Center, Sun Yat-sen University, Guangzhou, Guangdong, China

## Abstract

**Purpose:**

The present study highlighted the value of anterior segment optical coherence tomography (AS-OCT) for different types of corneal foreign bodies in humans.

**Methods:**

This study was a prospective observational study. The patients included were divided into two groups. If the patients were directly diagnosed based on eye injury history and slit-lamp examination, then they were assigned to Group A. Otherwise, the patients were assigned to Group B. We compared and described the characteristics of the corneal foreign body in both groups using AS-OCT.

**Results:**

From October 2017 to January 2020, 36 eyes of 36 patients (9 females and 27 males) with a mean age of 37.8 ± 11.7 years were included in the study. Patients in Group A were the majority and accounted for 72.2% (26/36). High signals on AS-OCT images were the main constituent and accounted for 92.3% (24/26) in Group A and 70.0% (7/10) in Group B. Most of the patients in Group A, 96.2% (25/26), had clear boundaries. A blurred boundary was observed in 70.0% (7/10) of the patients in Group B. The foreign bodies on AS-OCT images had key characteristics of a high signal followed by a central zone shadowing effect and a low signal followed by a marginal zone shadowing effect. Further, all of the lesions could be directly located in Group B, and 92.3% (24/26) of the patients in Group A did not have directly located lesions. Six representative cases are described in detail.

**Conclusions:**

AS-OCT is a valuable tool in the diagnosis and management of corneal foreign bodies, especially for unusual corneal foreign body.

## 1. Introduction

Corneal foreign bodies (FBs) are one of most common ophthalmological emergency cases. Patients with corneal foreign body (FB) have multiple ocular symptoms, including red eye, foreign body sensation, irritation, tearing, pain, and blurred vision. The timely and appropriate removal of a corneal FB is necessary [1–3]. Although the diagnosis and management of corneal FB is generally easily made based on the history and slit-lamp examination, there are some unusual cases of FB that create difficulties in the diagnosis and choosing most appropriate removal method due to the variety of FB [4–6].

Recently, optical coherence tomography (OCT), as a noninvasive examination technology, greatly improved the ophthalmologists' understanding and perception of diseases. The introduction of spectral-domain (SD) OCT technology and Fourier-domain (FD) OCT system led to a significant increase in acquisition speed and imaging resolution of the anterior segment [7, 8]. Anterior segment OCT (AS-OCT) generates detailed images and digital information on the anterior segment, and it was widely used to assess the phenotypes of the anterior segment of the eye such as corneal thickness and anterior chamber [9–11]. There were several case reports of corneal FBs using AS-OCT [12, 13] and observations of different corneal FB in vitro using AS-OCT [14]. However, no article indicates the diagnosis basis of cornea FB using AS-OCT.

The aim of the present study is to highlight the value of AS-OCT for the diagnosis of corneal FB and provide references for its management. In this study, we described the clinical findings and AS-OCT characteristics of different types of corneal FBs, compared the difference of two types of corneal foreign bodies, and pointed out a preliminary method for diagnosis of corneal foreign body using AS-OCT.

## 2. Materials and Methods

The present perspective observational study screened patients from October 2017 to January 2020 in our clinic with a diagnosed or suspected diagnosed corneal FB. All patients who were included this study met the following conditions:

1. Participated voluntarily in this study
2. Diagnosed or had a suspected diagnosis of cornea FB
3. Were able to complete the anterior segment examinations with clear anterior segment color photography and AS-OCT scanning
4. Had lesions with a depth that did not exceed 2/3 of the corneal thickness
5. Had no apparent corneal infection signs

Patients who did not meet any of the above criteria were not included in the study. This study adhered to tenets of the Declaration of Helsinki and was approved by the institutional review board and ethics committee from the Third Affiliated Hospital of Sun Yat-San University.

When the patient presented to our clinic, emergency treatment, such as topical anesthesia and flushing the conjunctival sac, was administered if necessary. Each patient underwent a careful, comprehensive eye examination. After slit-lamp examination, anterior segment color photography was taken, and the AS-OCT (3D OCT-2000, Topcon, Tokyo, Japan) scanner focused on lesions using the radial anterior segment protocol. The scan diameter was 6.0 mm, and a radial 12 hour scan was performed. One section of the clear images was used for used for evaluation and measurement.

In general, the patients with corneal FBs have clinical characteristics, such as a clear history of ocular trauma and ocular symptoms of red eye, foreign body sensation, irritation, tearing, pain, and blurred vision [3]. According to the clinical findings, we divided the patients into two groups (Group A and Group B). Three authors participated in the evaluation. Patients who could be diagnosed as corneal FB based on the medical history and anterior segment color photography were classified into Group A. If any of the three evaluators had doubts about the patient's diagnosis based on the medical history and the anterior segment color photography, the patient was classified into Group B.

We used the signal of the normal corneal tissue surrounding the lesions as a reference. If the signal of the lesions was stronger than the surrounding tissue signal, it was a hyperreflective signal (high signal). When the signal of the lesions was weaker than the surrounding tissue, it was a hyporeflective signal (low signal). The signal deep in the FB was weaker or even disappeared, which was regarded as signal attenuation and was called a shadowing effect [5, 7, 14].

Statistical data are described as frequencies and percentages for categorical data and means ± SD for numerical data using SPSS version 24 (SPSS Inc., Chicago, IL, USA).

## 3. Results

Thirty-six eyes of 36 patients (9 females and 27 males) with a mean age of 37.8 ± 11.7 years were included in the study.

Group A included 72.2% (26/36) patients, and Group B included 27.8% (10/36) patients. Metallic corneal FB was the most common observation and was observed in 65.7% (23/35) of all patients. In Group A, metallic corneal FB was observed in 88.5% (23/26) of patients, and only nonmetallic corneal FB were observed in Group B, 90.0% (9/10). One patient in Group B had a clear history of eye trauma and was eventually excluded from the diagnosis of corneal FB because of poor corneal epithelial healing.

Corneal FBs have some characteristics in AS-OCT images. A shadowing effect in the central zone behind the high signal and the marginal zone following the low signal was observed in both groups. FBs with high reflection signals were the most common in Group A (92.3%, 24/26) and Group B (70.0%, 7/10). Notably, the high and low signals of the FB corresponded to the central zone shadowing effect and marginal zone shadowing effect, respectively.

The location of corneal FBs is an important reference for selecting the removal ways of FBs. Increasing the depth and area of treatment will influence the time required to heal and may cause more visible corneal scars [3]. The selection of appropriate FB removal method, such as wiping, picking out, or scraping, is helpful to reduce corneal tissue damage and scar formation. The depth of the FB was generally indirectly located (92.3%, 24/26). However, whether there was a high or low signal in Group B, the location could be directly accomplished.

All descriptive characteristics of the study participants are summarized in Table 1.

To further demonstrate the value of AS-OCT in the diagnosis and management of different corneal FBs, six representative cases, including two cases in Group A and four cases in Group B, are reported in detail. The clinical details of each patient are presented in Table 2.

### 3.1. Case 1

**Figure 1:**
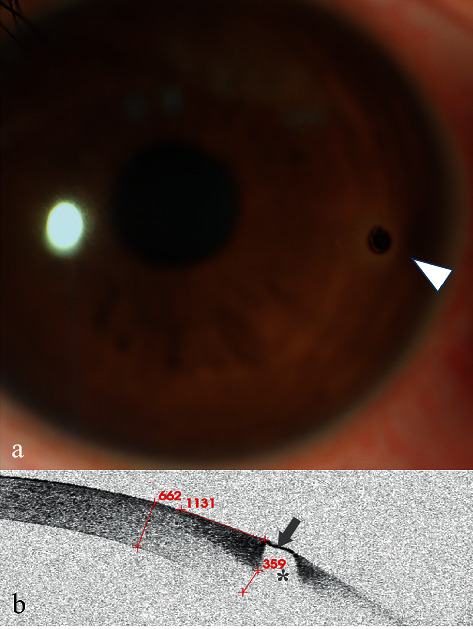
Anterior segment and AS-OCT image for Case 1. (a) Anterior segment photograph showed a corneal FB and inflammatory infiltration around the FB at an approximately 3:00 o'clock location approaching the limbus (arrowhead). (b) AS-OCT image showed that the FB showed a single high signal with clear boundary (arrow) following a central zone shadowing effect (star). The depth of the FB was estimated indirectly by subtracting the thickness of the underside of the high-reflected signal surrounding a FB (359 m) from the thickness of the cornea (662 m), which was less than 303 *μ*m.

A 31-year-old man presented to our clinic with redness in his left eye, photophobia, and foreign body sensation. He used an electric saw to cut iron two days prior. Slit-lamp examination revealed an iron FB surrounded by a slight haze (Figure 1(a), arrowhead). Corneal FB was easily diagnosed. The FB was removed via picking and scraping.

AS-OCT scanning showed a single high signal with clear boundary (Figure 1(b), arrowhead) following by central zone shadowing effect (Figure 1(b), star). The depth of the FB could not be directly located.

### 3.2. Case 2

**Figure 2:**
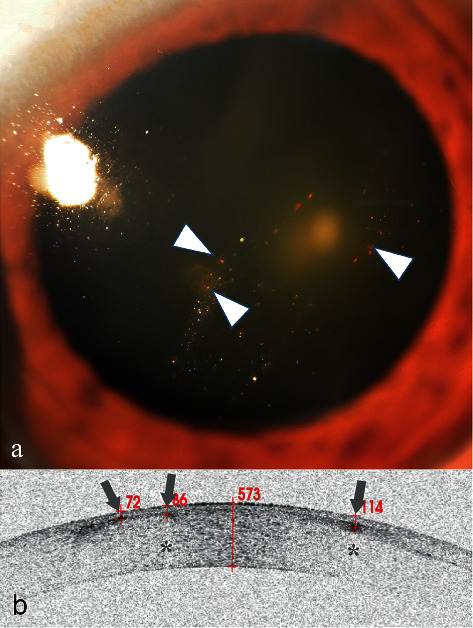
Anterior segment and AS-OCT image for Case 2. (a) Anterior segment photograph showing multiple small granular corneal lesions in the shallow cornea (arrowheads). (b) AS-OCT showed that there were three high signals with blurred boundary (arrows). Two of the signals had a central zone shadowing effect (stars). They were directly located and were 72, 86, and 114 *μ*m beneath the corneal surface.

A 69-year-old man presented with itching eyes for several weeks. Slit-lamp examination revealed multiple granular corneal opacities (Figure 2(a), arrowheads) in his left cornea. Corneal fluorescein staining was negative. The patient remembered that his left eye was injured by a tire explosion one decade ago. An old multiple corneal FB diagnosis was concluded. Because of the long harmless history, these FBs were not further treated.

AS-OCT scanning showed some high signals with blurred boundary (Figure 2(b), arrows) followed by a central zone shadowing effect (Figure 2(b), stars) in the deep part of the epithelium and the superficial portion of the stroma. The depth of the FB was directly located (Figure 2(b)).

### 3.3. Case 3

**Figure 3:**
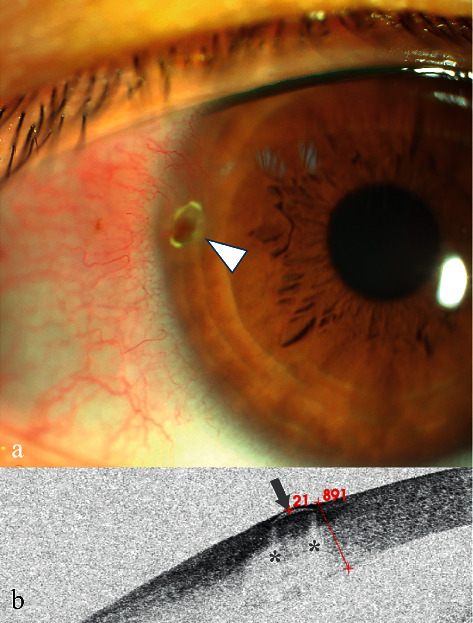
Anterior segment and AS-OCT image for Case 3. (a) Anterior segment photograph: at 9:00 o'clock inside the limbus (arrowhead), a light brown mass was observed without surface fluorescence staining, and fluorescein gathered around the edges. Neovascularization was also seen. (b) AS-OCT image showing that a crescent-shaped low reflective signal (arrowhead) with bilateral marginal zone shadowing (stars) was found 21 *μ*m below the epithelium surface.

A 30-year-old woman presented with redness in her right eye that lasted for two weeks. She experienced no other discomfort. Examination revealed a light brown mass on the temporal limbus of her right cornea (Figure 3(a), arrowhead), and vascularization was observed in the center of the lesion. Corneal fluorescein staining was negative on the mass surface. On further questioning, her history revealed that a FB may have been blown into her eye one month ago, but she reported no discomfort, and no treatment was administered. Her diagnosis was a suspected corneal FB, and corneal neoplasm could not be excluded.

AS-OCT scanning showed a single low signal with clear boundary (Figure 3(b), arrow) followed by a marginal zone shadowing effect (Figure 3(b), stars). The depth of the FB was directly located. The FB was picked out using a needle and was confirmed as a translucent shell-like FB.

### 3.4. Case 4

**Figure 4:**
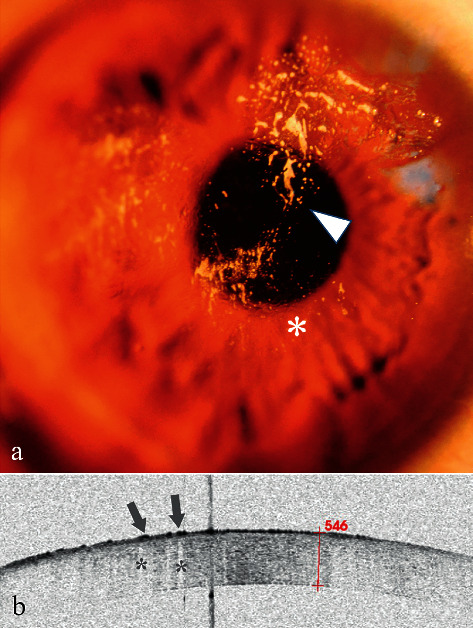
Anterior segment and AS-OCT image for Case 4. (a) Anterior segment photograph showing multiple cream-like corneal opacities (arrowheads) surrounding a wrinkled transparent cornea. (b) AS-OCT image showed multiple spines (high signals) with blurred boundary (arrows) on the superficial epithelium followed by a central zone shadowing effect (stars). The corneal thickness was 546 *μ*m, within the normal range.

A 39-year-old man presented to the emergency room because glass glue was splashed into his right eye 30 minutes prior. After emergency treatment, he was further examined. Slit-lamp examination revealed cream-like particulates (Figure 4(a), arrowhead) and microfolds on the cornea (Figure 4(a), star). His diagnosis was not clear because his corneal degeneration may be due to chemical injuries, FB of residual glue, or both.

AS-OCT examinations revealed multiple high signals with blurred boundary (Figure 4(b), arrows) followed by a central zone shadowing effect (Figure 4(b), stars). The lesions were located on the corneal surface. The corneal thickness was in the normal range of 546 *μ*m, and the corneal stromal signal was intact. The diagnosis was corneal chemical injury (corneal epithelial degeneration) and corneal FB (glass glue). Because the lesions were located in a shallow and wide range on the cornea, wiping the FB with wet swabs was used for treatment. No other damage to the cornea was found during the follow-up days.

### 3.5. Case 5

**Figure 5:**
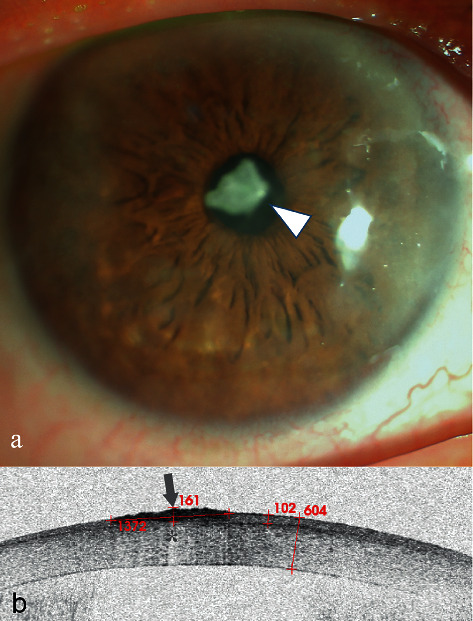
Anterior segment and AS-OCT image for Case 5. (a) Anterior segment photograph showing a patchy cloud-like haze over the cornea and a cloudy milky lump in front of the pupil (arrowhead). (b) AS-OCT scanning showed there was a lesion of high signal with blurred boundary (arrow) followed by a central zone shadowing effect (star). The lesion had a diameter of 1372 *μ*m. The corneal thickness was 604 *μ*m, and the epithelial thickness was 102 *μ*m. A layer of continuous epithelium tissue was seen on the bottom of the lesion.

A 62-year-old man with blurred vision in his right eye for six months was diagnosed with viral keratitis at his local hospital and continuously received antiviral therapy. He denied a history of eye injuries. Slit-lamp showed a white lump on the cornea in the pupil region (Figure 5(a), arrowhead). His diagnosis was drug-induced keratitis with a suspected corneal FB.

AS-OCT revealed a high signal with blurred boundary (Figure 5(b), arrowhead) in the epithelial layer, under which was a partially continuous epithelium. It was followed by a central zone shadowing effect (Figure 5(b), star). The lesions were scraped with a needle. On the following day, his cornea was restored to nearly transparent, and his visual acuity went from 20/100 back to 20/25.

### 3.6. Case 6

**Figure 6:**
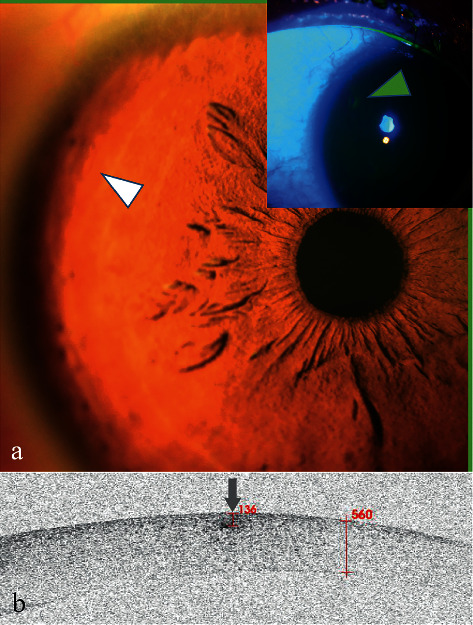
Anterior segment and AS-OCT image for Case 6. (a) Anterior segment photograph showed a slight bulge at 10:00 o'clock in the peripheral cornea (white arrowhead). Corneal fluorescein staining was negative (arrowhead). (b) AS-OCT scanning showed there was a lesion of high signal with blurred boundary (arrow) without a shadowing effect. The lesion was directly located and was 136 *μ*m beneath the corneal surface.

A 28-year-old man complained of a constant foreign body sensation in his right eye. His right eye was cut by his broken glasses approximately 2 weeks prior. Careful inspection with the slit-lamp revealed a slight bulge in the peripheral cornea (Figure 6(a), white arrowhead). Corneal fluorescein staining was negative (Figure 6(a), green arrowhead). His diagnosis was a suspected corneal FB.

AS-OCT scanning showed a high signal with blurred boundary (Figure 6(b), arrow), but there was no shadowing effect beneath the lesion. It was directly located and was 136 *μ*m in the cornea. Because of the poor epithelial healing in the lesion area that caused the foreign body sensation, scraping the epithelium of the lesion area was chosen. There was no FB to be further confirmed.

## 4. Discussion

From this study, we can obtain a method of using AS-OCT to diagnose corneal foreign body. In addition, through the localization of corneal foreign body, a more appropriate means of removing corneal foreign body is selected. To the best of our knowledge, this study is the first article that indicates the diagnosis basis of cornea FB using AS-OCT.

AS-OCT clearly showed the fine structure of the cornea [15–17]. The presence of a FB damages the consistency of the corneal structure, which provides the basis for the diagnosis of a FB. FBs show high or low signals on AS-OCT images. High signals followed by a shadowing effect were reported in previous literature [5, 14, 18]. However, these studies ignored the phenomenon that the low signals could also be followed by a shadowing effect, which we named a marginal zone shadowing effect. We hypothesized that this effect may be a total reflection phenomenon resulting from the rupture of the corneal tissue around the FB.

Comparing the AS-OCT characteristics of two groups, we could develop the diagnostic criteria for corneal FB using AS-OCT as follows:

1. Eye injury history
2. The consistency of the corneal layers being broken
3. High or low signals with clear boundaries
4. High or low signals with blurred boundaries
5. Lesions associated with central or marginal zone shadowing effect

If conditions (1) + (2) + (3) + (5) were met, then a corneal FB was diagnosed.

If conditions (1) + (2) + (4) + (5) were met, then a corneal FB was highly suspected.

When conditions (1) and (2) are met, and there is a high signal with blurred boundary but a lack of condition (5), the diagnosis of corneal FB using AS-OCT should be fairly cautious. The local inflammation, corneal scarring, or corneal neoplasm should be investigated [19–24].

AS-OCT scanning may also help optimize the removal of FB. The choice of a suitable corneal FB removal method helps reduce the corneal damage and the formation of corneal scars. According to the location of the corneal FB on the AS-OCT, especially in Group B, we selected different methods to minimize the damage to the cornea and reduce the risk of visual impairment. For example, if the FB was located in the epithelial layer and mobile, and Bowman's layer was untouched, wiping methods were preferred to avoid the forming of a corneal scar. With the application of AS-OCT technology, the frequency of patients' visits may be reduced.

The present study has some limitations. First, the number of subjects we studied was relatively small compared to the various types of corneal FB. Second, there were not enough negative controls, such as corneal infection or degeneration, to further validate the criteria. Therefore, the diagnostic method we summarized need more cases to continue improvements. Third, we used 3D OCT-2000 for AS-OCT scanning in this study, which is a spectral-domain optical coherence tomography developed for the ocular fundus. Although AS-OCT with the cornea anterior module was functional, the AS-OCT observation of corneal FB was hampered by a number of limitations.

In summary, the results of the present study suggest that AS-OCT is a very valuable tool for the diagnosis of corneal FB, especially for some unusual cases. AS-OCT scanning could provide a reference for the selection of FB management through direct or indirect FB localization. The value of AS-OCT application in the corneal FB should be further strengthened.

## Figures and Tables

**Table 1:** Clinical characteristics of all patients.

	Group A	Group B
Gender (F/M)	6/20	3/7
Age (years)	38.4 ± 11.5	36.1 ± 12.0
Metallic FB	23	0
Nonmetallic FB	2	9
Mixed FB	1	0
H signal	24	7
L signal	2	3
Clear boundary	25	3
Blurred boundary	1	7
Central shadowing	24	6
Marginal shadowing	2	3
Direct location	2	10
Indirect location	24	0

**Table 2:** Clinical details and AS-OCT characteristics of cases.

Case no.	1	2	3	4	5	6
Group	A	A	B	B	B	B
Sex/age	M/31	M/69	F/30	M/39	M/62	M/28
Numbers of FB	Single	Multiple	Single	Multiple	Single	None
Nature of FB	Metallic	Mixed	Nonmetallic	Nonmetallic	Nonmetallic	^*∗*^
Reflective signal	High	High	Low	High	High	High
Border of lesions	Clear	Unclear	Clear	Unclear	Unclear	Unclear
Shadowing effect	Central	Central	Marginal	Central	Central	Negative
Location depth	Indirect	Direct	Direct	Direct	Direct	Direct

^*∗*^The case eventually proved to be free of FBs.

## Data Availability

The data used to support the findings of this study are available from the corresponding author upon request.
